# NEIGHBORHOOD WALKABILITY INFLUENCES ASSOCIATIONS BETWEEN PHYSICAL ACTIVITY AND GAIT MEASURES IN OLDER ADULTS

**DOI:** 10.1093/geroni/igac059.1012

**Published:** 2022-12-20

**Authors:** Anisha Suri, Xiaonan Zhu, Geeta Acharya, Alyson Harding, Jessie VanSwearingen, Mark Redfern, Ervin Sejdic, Andrea Rosso

**Affiliations:** Swanson School of Engineering, University of Pittsburgh, Pittsburgh, Pennsylvania, United States; University of Pittsburgh, Pittsburgh, Pennsylvania, United States; University of Pittsburgh, Pittsburgh, Pennsylvania, United States; University of Minnesota, Minneapolis, Minnesota, United States; School of Health and Rehabilitation Sciences, Pittsburgh, Pennsylvania, United States; Swanson School of Engineering, University of Pittsburgh, Pittsburgh, Pennsylvania, United States; University of Toronto, Toronto, Ontario, Canada; University of Pittsburgh, Pittsburgh, Pennsylvania, United States

## Abstract

Neighborhood walkability can influence physical activity and of older adults. Residential neighborhoods of participants (N=186, 77±6 years, 70% females) were audited for walkability using Google Street View. Factor analysis categorized neighborhood walkability as high, medium, and low. Gait quality was derived from a 4-m instrumented walkway (pace, variability, walk-ratio) and accelerometry signals at the lower back during a 6-minute walk test (adaptability, similarity, and smoothness). Activity was step-count from seven-day actigraphy. We studied associations between gait variables and step-count across high, medium, and low walkability neighborhoods using linear regression (age and sex as covariates). Pace(m/s) [High(β=0.46, p<.05), Medium(β=0.43, p<.05), Low(β=0.25, p>.05)], adaptability(m/s2) [High(β=0.47, p<.05), Medium(β=0.24, p<.05), Low(β=0.37, p<.05)], and similarity [High(β=0.39, p<.05), Medium(β=0.28, p<.05), and Low(β=0.18, p>.05)] were associated with step-count, stronger associations for high walkability neighborhoods (p for interactions <0.01). No associations with variability, walk-ratio, and smoothness were found. Associations between gait and activity differed by neighborhood walkability.

